# Supplementary material for: Splice-Junction-Based Mapping of Alternative Isoforms in the Human Proteome
Source: Cell Rep. Author manuscript; Available in PMC 2020 Jan 15. (PMC6961840; doi:10.1016/j.celrep.2019.11.026)
Supplement: 3 [file NIHMS1546469-supplement-3.zip › DF2/PXD0010154/Lung-74-Q7KZI7-DSTAPQRVPVASPSAHNISSSGGAPDR.pdf]

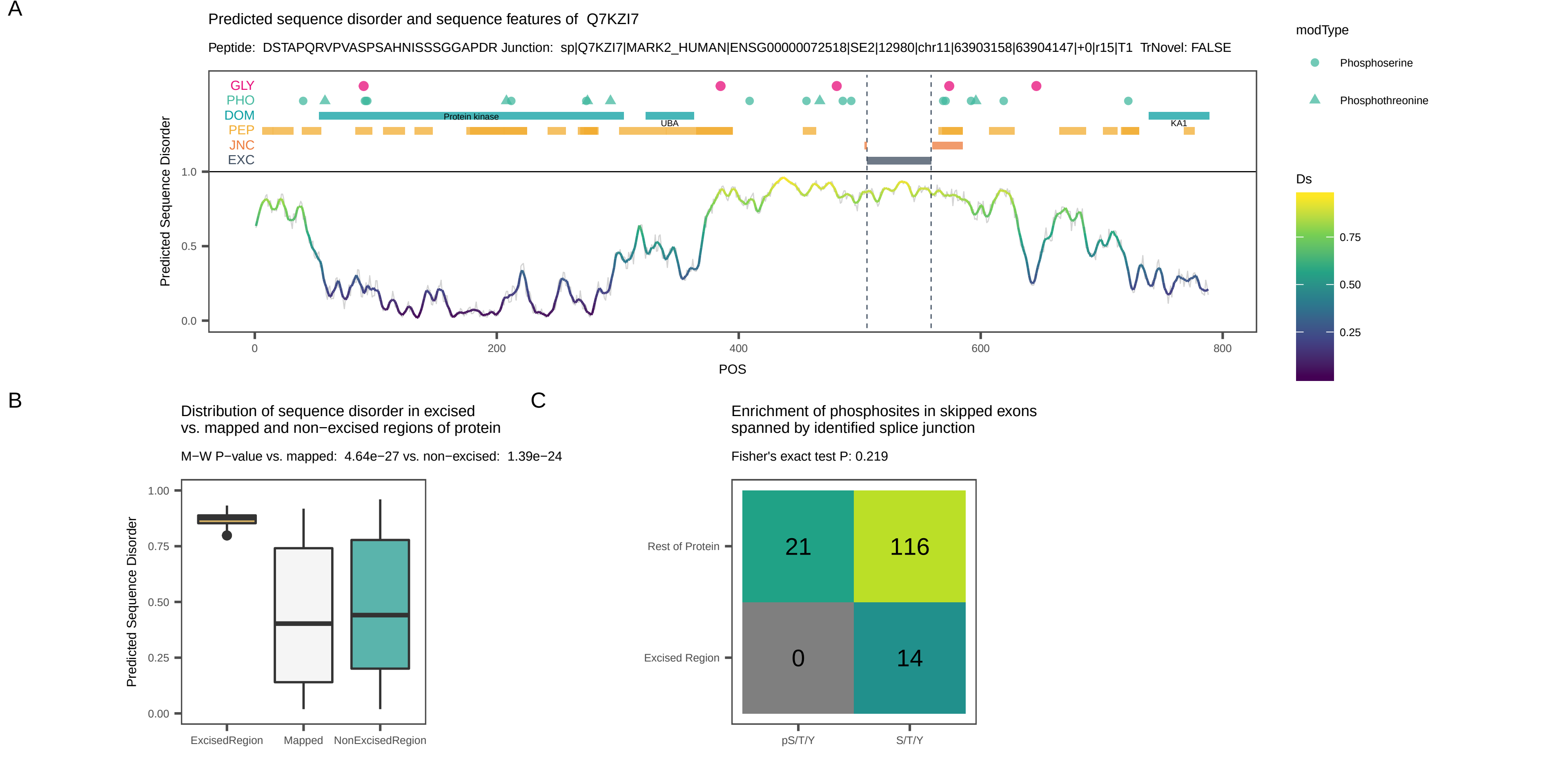
A
Predicted sequence disorder and sequence features of Q7KZI7
Peptide: DSTAPQRVPVASPSAHNISSSGGAPDR Junction: sp|Q7KZI7|MARK2_HUMAN|ENSG00000072518|SE2|12980|chr11|63903158|63904147|+0|r15|T1 TrNovel: FALSE
GLY
PHO
DOM
PEP
JNC
EXC
Protein kinase
UBA
KA1
Predicted Sequence Disorder
POS
modType
Phosphoserine
Phosphothreonine
Ds
B
Distribution of sequence disorder in excised vs. mapped and non−excised regions of protein
M−W P−value vs. mapped: 4.64e−27 vs. non−excised: 1.39e−24
ExcisedRegion
Mapped
NonExcisedRegion
C
Enrichment of phosphosites in skipped exons spanned by identified splice junction
Fisher's exact test P: 0.219
Rest of Protein
Excised Region
pS/T/Y
S/T/Y
21
116
0
14
